# Draft Genome Sequence of a New Bacterium Named *Rappaport israeli*, gen. nov., sp. nov., Isolated from a Blood Culture

**DOI:** 10.1128/genomeA.01595-16

**Published:** 2017-02-09

**Authors:** Hiba Waldman Ben-Asher, Rebecca Yerushalmi, Chaim Wachtel, Efrat Barbiro-Michaely, David Sompolinsky, Doron Gerber

**Affiliations:** a Everard and Mina Goodman Faculty of Life Sciences, Bar-Ilan University, Ramat Gan, Israel; b Laboratory of Microbiology, Mayanei HaYeshua Medical Center, Bnei Brak, Israel

## Abstract

Here, we report the draft genome sequence of a Gram-negative microbe found in a blood culture (B08008) from a patient. The organism was proposed to be from a new unknown genus and species. This publication will increase worldwide microbial knowledge and may improve microbial identification and antibiotic treatment for patients.

## GENOME ANNOUNCEMENT

The new *Rappaport israeli* bacterium was isolated from a blood culture (B08008) from a 4-year-old patient hospitalized at Mayanei-HaYeshua Medical Center in Israel. *De novo* sequencing of the new *Rappaport israeli* bacterium was conducted at the Bar-Ilan University Microarray Unit, Ramat Gan, Israel. DNA libraries were generated as follows. DNA was extracted from 2 ml of culture using the DNeasy blood and tissue kit (Qiagen), as per the manufacturer’s instructions. For each sample, 100 μg of DNA was used to prepare a barcoded library for sequencing, using the Ion Xpress Plus fragment library kit (Life Technologies, Inc.). Briefly, DNA was fragmented with Ion Shear enzyme mix II, and adaptors and barcodes were ligated to the fragmented DNA. The ligated DNA was then size selected on a 2% E-Gel SizeSelect agarose gel (Invitrogen). The size-selected library was then amplified using the primers supplied in the Ion Xpress Plus fragment library kit (Life Technologies, Inc.). The concentrations of the libraries were measured using a Qubit double-stranded DNA (dsDNA) high-sensitivity (HS) assay kit, and the size distribution was analyzed with an Agilent high-sensitivity DNA kit. Libraries were prepared for sequencing on a One Touch 2 using the Ion PGM Template OT2 200 kit (Life Technologies, Inc.). The sequencing was then performed on an Ion Torrent PGM using Ion PGM sequencing 200 kit version 2 (Life Technologies, Inc.).

An overall 1.5 million reads (exact number = 1,564,745) with a mean length of 185 bp were received. Data were analyzed using the *de novo* Newbler assembler (Roche). The final draft genome sequence comprises 43 contigs (longer than 500 bp), with a mean size of 40,607 bp and a maximum length of 286,678 bp. The total length of the genome was 1,751,693 bp, with a mean G+C content of 45% and an average coverage of approximately 186×. Contig annotation was conducted using the Rapid Annotations using Subsystems Technology (RAST) server (1).

The final draft genome sequence contains 2,178 coding sequences (of which 1,529 possess annotated functions and 649 are hypothetical proteins) and 40 RNA genes.

### Accession number(s).

This whole-genome shotgun project has been deposited at DDBJ/ENA/GenBank under the accession no. LXHZ00000000. The version described in this paper is version LXHZ01000000.
